# Salbutamol Attenuates Diabetic Skeletal Muscle Atrophy by Reducing Oxidative Stress, Myostatin/GDF-8, and Pro-Inflammatory Cytokines in Rats

**DOI:** 10.3390/pharmaceutics15082101

**Published:** 2023-08-08

**Authors:** Anand Kumar, Priyanka Prajapati, Gurvinder Singh, Dinesh Kumar, Vikas Mishra, Seong-Cheol Kim, Chaitany Jayprakash Raorane, Vinit Raj, Sapana Kushwaha

**Affiliations:** 1 Department of Pharmaceutical Sciences, School of Pharmaceutical Sciences, Babasaheb Bhimrao Ambedkar University, Vidya Vihar, Raebareli Road, Lucknow 226025, India; anandkumarpharm@gmail.com (A.K.); priyankaprajapati243@gmail.com (P.P.); vikasmishra12@gmail.com (V.M.); 2 Centre of Biomedical Research, SGPGIMS Campus, Lucknow 226014, India; gourav1752@gmail.com (G.S.); dineshcbmr@gmail.com (D.K.); 3 School of Chemical Engineering, Yeungnam University, Gyeongsan 38541, Republic of Korea; sckim07@ynu.ac.kr; 4 National Institute of Pharmaceutical Education and Research (NIPER), Raebareli, New Transit Campus, Bijnor-Sisendi Road, Lucknow 226002, India

**Keywords:** diabetes, salbutamol, skeletal muscle atrophy, sarcosine, metabolomics

## Abstract

Type 2 diabetes is a metabolic disorder that leads to accelerated skeletal muscle atrophy. In this study, we aimed to evaluate the effect of salbutamol (SLB) on skeletal muscle atrophy in high-fat diet (HFD)/streptozotocin (STZ)-induced diabetic rats. Male Sprague Dawley rats were divided into four groups (*n* = 6): control, SLB, HFD/STZ, and HFD/STZ + SLB (6 mg/kg orally for four weeks). After the last dose of SLB, rats were assessed for muscle grip strength and muscle coordination (wire-hanging, rotarod, footprint, and actophotometer tests). Body composition was analyzed in live rats. After that, animals were sacrificed, and serum and gastrocnemius (GN) muscles were collected. Endpoints include myofibrillar protein content, muscle oxidative stress and antioxidants, serum pro-inflammatory cytokines (interleukin-1β, interleukin-2, and interleukin-6), serum muscle markers (myostatin, creatine kinase, and testosterone), histopathology, and muscle ^1^H NMR metabolomics. Findings showed that SLB treatment significantly improved muscle strength and muscle coordination, as well as increased lean muscle mass in diabetic rats. Increased pro-inflammatory cytokines and muscle markers (myostatin, creatine kinase) indicate muscle deterioration in diabetic rats, while SLB intervention restored the same. Also, Feret’s diameter and cross-sectional area of GN muscle were increased by SLB treatment, indicating the amelioration in diabetic rat muscle. Results of muscle metabolomics exhibit that SLB treatment resulted in the restoration of perturbed metabolites, including histidine-to-tyrosine, phenylalanine-to-tyrosine, and glutamate-to-glutamine ratios and succinate, sarcosine, and 3-hydroxybutyrate (3HB) in diabetic rats. These metabolites showed a pertinent role in muscle inflammation and oxidative stress in diabetic rats. In conclusion, findings showed that salbutamol could be explored as an intervention in diabetic-associated skeletal muscle atrophy.

## 1. Introduction

Type 2 diabetes mellitus (T2DM) is a metabolic disorder that causes elevated blood glucose levels due to compromised insulin function and/or release [1]. Diabetes poses a significant global health challenge, with far-reaching implications that can impact patients’ lives and well-being [2]. The interventions and management of microvascular (retinopathy, nephropathy, and neuropathy) and macro-vascular (cardiomyopathy) problems associated with diabetes are key goals of care for diabetic patients [3,4]. Diabetes is associated with diabetic myopathy, resulting in reduced skeletal and muscular strength [1,5,6]. However, this common condition lacks adequate research attention. It poses a significant clinical challenge, leading to a poorer quality of life for affected individuals. Skeletal muscle, constituting over 40% of total body mass in men and around 30% in women, is the predominant component in the human body [7]. Skeletal muscle is involved in a broad spectrum of physiological activities, such as metabolism, thermogenesis, and protein synthesis. These functions enable it to establish connections with other tissues, support an upright posture, and facilitate movement throughout the body [8]. Being the largest organ, skeletal muscle plays a crucial role in regulating glucose levels in the body. During the postprandial stage, it serves as a primary site for insulin-stimulated glucose absorption, facilitated by the translocation of glucose transporter type 4 (GLUT4) [9]. Oxidative stress and mild persistent inflammation are essential underlying causes of diabetic muscle dysfunction [1,10,11]. During oxidative stress and chronic inflammation, a multitude of intracellular signaling pathways undergo activation or inactivation [11]. This process results in detrimental effects such as apoptosis, impaired muscle progenitor cells (muscle satellite cells), and compromised myogenic capacity. Additionally, extracellular matrix (ECM) remodeling plays a pivotal role in the primary pathology, contributing to substantial muscle mass loss [1]. Findings suggest that oxidative stress and increased levels of transforming growth factor-β and tumor necrosis factor-α enhance ubiquitin proteolytic activity via specific E3 ubiquitin-ligase genes, including F-box-only protein 32 (FBXO32) and muscle-specific RING finger protein 1 (MuRF-1). This process leads to reduced protein synthesis and increased degradation of proteins in various skeletal muscles, including those affected by diabetic myopathy [12,13,14].

Salbutamol, known as albuterol, is a short-acting synthetic medication that is selective to β2-adrenoceptors (βAR). It is primarily used as a bronchodilator to manage bronchial asthma and chronic obstructive pulmonary disease (COPD). In previous research, it was observed that β2-adrenoceptors (βAR) and cyclic adenosine monophosphate (cAMP) mediated through the ubiquitin-proteasome system (UPS) [15]. Additionally, the selective β2-agonist clenbuterol, which belongs to the same drug category as salbutamoldemonstrated an anti-proteolytic effect during food deprivation. This effect is mediated through a cAMP/Akt-dependent pathway, leading to Foxo3a phosphorylation, suppression of atrogin-1, and inhibition of ubiquitination [16,17]. The study showed that short-term use of salbutamol increased voluntary muscle strength in humans [18]. However, the effect varied among different muscle groups. These findings suggest the therapeutic potential of β2-adrenoceptor agonists in modulating skeletal muscle function in humans. Both salbutamol and clenbuterol were administered to rats of different ages for three weeks, resulting in increased weight and protein content of hind-limb muscles in young and old rats. Additionally, both drugs increased the protein content of the whole body (carcass) and promoted muscle protein recovery in senescent rats [19]. Salbutamol has been found to enhance protein turnover rates in skeletal muscle after exercise in human subjects [20]. This effect involves the activation of cAMP/PKA and Akt2 signaling pathways and modulation of mRNA expression of growth-regulating proteins. These findings exhibit that salbutamol has the potential to enhance muscle protein synthesis and promote muscle growth in response to exercise [20]. Interestingly, a two-week course of salbutamol administration improved abilities in performing repeated sprints, physical performance, and muscle strength in athletic individuals [20]. Furthermore, salbutamol stimulates the transformation of muscle fiber isoforms, specifically myosin-heavy-chain (MHC)-I to MHC IIa, and enhances the hypertrophy of MHC IIa fibers after weight exercise [21]. A recent clinical trial demonstrates that albuterol (salbutamol) improved muscle function in Pompe disease and showed potential benefit as an adjunctive treatment along with enzyme replacement therapy [22].

Taken together, these promising findings suggest that salbutamol could be repurposed for the treatment of muscle atrophy. Based on these findings, we hypothesized that salbutamol might provide protection against skeletal muscle atrophy in diabetic rats induced by a high-fat diet (HFD)/streptozotocin (STZ). Furthermore, studies suggest that specific metabolites identified through plasma and skeletal muscle metabolomics profiles of well-phenotype diabetes patients may play a role in the pathophysiological pathway leading to the development of type 2 diabetes [23,24]. ^1^H nuclear magnetic resonance (NMR) is a new technique used to detect these metabolites in the muscle tissue of diabetic patients [25] and enables researchers to provide insights into the metabolic disruptions in diabetic skeletal muscle. We further investigated how salbutamol modulates these altered metabolites in the skeletal muscle of diabetic rats.

## 2. Materials and Methods

### 2.1. Chemicals and Reagents

Salbutamol was received as a gift sample from Cipla Ltd., Mumbai, India. All the chemicals used in the present study were purchased from Sigma, St. Louis, MO, USA, and MP Biomedicals, Santa Ana, CA, USA, unless specified. The ELISA kits, IL-6 (Cat#550319) was purchased from BD Biosciences, San Jose, CA, USA. IL-2 ELISA kit (Cat# RAB0288) kit was purchased from Sigma, St. Louis, MO, USA. IL-1β (Cat #E-EL-R0012) kit was purchased from Elab Biosciences, Houston, TX, USA. Creatine kinase (Cat #3100709) and testosterone (Cat #3110023) ELISA kits were purchased from Real Gene Labs, Los Angeles, CA, USA GDF-8 ELISA kit (Cat #DGDF80) was purchased from the R&D System, Minneapolis, MN, USA. LiquiMax HDL Direct, LDL Cholesterol Direct, LiquiMax Cholesterol, Triglycerides kits were purchased from Avecon Health Care Pvt. Ltd., Haryana, India. High-fat diet was purchased by Bharat Ansh Scientific Industries, Lucknow, India. All the solvents used in the present study were of analytical grade.

### 2.2. Experimental Design

The animal experiments were performed according to the Committee for Control and Supervision of Experiments on Animals (CCSEA) guidelines approved by the Institutional Animal Ethical Committee (IAEC) of Babu Banarasi Das Northern India Institute of Technology (BBDNIIT) Lucknow, India (IAEC approval no. BBDNIIT/IAEC/2019/19). Twenty-four male Sprague Dawley (SD) rats aged 8–10 weeks and weighing 200 ± 30 g were purchased from the Central Drug Research Institute (CDRI), Lucknow, India. Standard laboratory conditions were maintained (22 ± 4 °C), and the animals were kept under an ambient environment (12 h light/dark cycles), humidity (50–60%), and water ad libitum. Animals were housed in polypropylene cages, and one week before starting the experiment, the rats were acclimatized to workable conditions. Twenty-four rats were allocated and randomized into four groups (*n* = 6): group I (control) received citrate buffer as a vehicle; group II (salbutamol, per se) was the control group treated with 6 mg/kg salbutamol (SLB) orally for four weeks once daily; group III (HFD/STZ) had a high-fat diet ad libitum for two weeks and injected as a single low dose of 35 mg/kg of streptozotocin (STZ) intraperitoneally (i.p.) (hereafter named as HFD/STZ group); and group IV served as HFD/STZ + salbutamol. SLB was prepared in distilled water at administered 6 mg/kg per oral for four weeks once daily. Blood samples were collected from the retro-orbital plexus under light anesthesia, and serum was separated and stored at −20 °C for further endpoints. Rats were sacrificed humanely by cervical dislocation, and the gastrocnemius muscle (GN) was isolated and preserved in 10% formalin for histology and kept at −80 °C for other endpoint parameters. The gastrocnemius (GN) muscle is chosen for its unique composition of both type 1 and type 2 muscle fibers. Additionally, it has been extensively studied in previous research [26,27].

#### 2.2.1. Induction of Type 2 Diabetes

The Srivansan et al. model was used for the induction of type 2 diabetes [28]. In brief, SD male rats were given ad libitum access to a high-fat diet (HFD), having fat (58%), protein (25%), and carbohydrate (17%) for two weeks. After the initial two weeks of HFD diet, a single intraperitoneal injection of a low dose of STZ (35 mg/kg) was administered, and the animals were then continued on the HFD feeding for an additional two weeks. At the end of the four weeks, fasting plasma glucose, insulin, and lipid profile (triglyceride and cholesterol) were measured to confirm the induction of type-2 diabetes. Rats with fasting plasma glucose levels of ≥250 mg/dL or higher were considered diabetic and were used in the present experiments. The STZ was prepared in a citrate buffer (pH of 4.5, while the respective control rats were given a vehicle (citrate buffer) in volume of 1 mL/kg, intraperitoneally. Blood glucose levels were recorded with a glucometer (Dr Morepen GlucoOne, model-BG3).

#### 2.2.2. Rationale of Selection of Salbutamol Dose

Based on an extensive literature review, it was observed that salbutamol is commonly administered in microgram doses, primarily in studies focusing on asthma. Furthermore, two findings were identified that repurposed salbutamol for different research purposes, specifically sepsis [29] and in the central nervous system [30]. In these studies, rats were administered salbutamol at doses of 4 mg/kg and 10 mg/kg for specific research objectives. Based on these findings, we carefully selected a dose within the range of these two studies. Therefore, in the present study, we chose a dose of 6 mg/kg of salbutamol.

### 2.3. Estimation of Body Weight, Gastrocnemius (GN) Muscle Weight, and Blood Glucose Levels

The body weight and gastrocnemius muscle weight of the rats were weighed using an analytical balance at the beginning and end of the experiment. Next, blood glucose levels were assessed using a glucometer at the start and end of the four-weeks experiment.

### 2.4. Estimation of Body Composition

The body composition of rats, specifically lean mass and fat mass, was assessed using the EchoMRI-500 body composition analyzer (EchoMRI Corporation Pvt. Ltd., Singapore). All the animals were gently placed in a specially designed, clear plastic holder without the need for anesthesia. The holder was then inserted into a designated tubular space on the side of the EchoMRI™ machine. By pressing a key on the keyboard, we initiated the scanning process and recorded the values for fat mass (in grams), lean body mass (in grams), free water (in grams), total water (in grams), and body weight (in grams) of each rat. The body weight, fat mass, and lean body mass were extracted from the collected data. These parameters were utilized to calculate the percentage change in lean mass and fat mass. These calculations were carried out to analyze the body composition, with a specific focus on the distribution of lean and fat mass. The data were expressed in percentages [31,32].

### 2.5. Behavioral Parameters

#### 2.5.1. Assessment of Forelimb Grip Strength by Grip Strength Meter

Rats were raised by their tails and forced to grab a hard bar connected to a mechanical force sensor of grip strength meter (LEGSM-01; Milton Enterprises, Nashik, India). Each rat was gently dragged backward by the tail, and grip strength was determined by tension reading on the digital force gauge shortly before the rat let go of the bar [33,34].

#### 2.5.2. Assessment of Locomotor Activity by Actophotometer Test

Random locomotor activity was measured using an actophotometer. Each animal was monitored for 5 min in a 14 × 14 × 14 cm^2^ closed-field arena with six photocells on the outside wall. A six-digit counter was used to track the photocell light interruptions (locomotor activity). The actophotometer was turned on, and each rat was placed in the cage independently for 5 min to measure locomotor activity [35].

#### 2.5.3. Assessment of Muscle Strength by Wire-Hanging Test

The hanging wire analysis was performed to determine the coordination and muscle strength of the rats. It involves suspending the rat from a one-meter height at a stainless-steel wire of approximately 2.5 cm width over a soft fall area for three minutes. The number of falls of each animal was recorded using a digital camera with a cut-off of 10 falls [36].

#### 2.5.4. Assessment of Muscle Coordination by Rotarod Test

The rotarod instrument assessed motor coordination and balance in the forelimbs and hind limbs. The latency to fall was determined after each rat was placed on the rotarod for 5 min at a constant speed of 5 rpm during the trial phase. Each experiment included three runs. Each rat was placed on the rotarod for a maximum of 5 min at a speed that increased from 4–40 rpm during the test, and the latency to fall was recorded [35].

#### 2.5.5. Assessment of Gait Speed by Footprint Test

At the end of the experiment, the gait cycle of the respective groups (control, salbutamol, HFD/STZ, and HFD/STZ + salbutamol) animals was assessed using the footprint test. The forefeet and hind feet of the different groups rats (control, salbutamol, HFD/STZ, and HFD/STZ + salbutamol) were painted with different non-toxic colors to obtain footprints. Subsequently, the rats were allowed to walk on white paper. The average distance moved forward between the steps was used to compute the stride length. The average distance between the left and right hind footprints was used to calculate the width of the hind and front bases. To assess step alternation homogeneity, the distance between the left-to-left and right-to-right front footprint/hind footprint overlap was measured. For the assessment, a set of six steps was chosen, eliminating impressions created at the start and end of the run [37].

### 2.6. Estimation of Total and Myofibrillar Protein Concentration

Total and myofibrillar proteins from rats GN skeletal muscles have been estimated as per previous protocol [38]. In brief, 50 mg of GN muscles were homogenized on ice-cold buffer, pH 6.8, containing sucrose (8.5%), EDTA (5 mM), KCl (50 mM), and MgCl_2_ (100 mM). This homogenate was used to measure the concentration of total protein. Further, GN homogenate was centrifuged at 2500× *g* for 15 min at 4 °C. The supernatant was then discarded, and the remaining pellet was re-suspended in a solution (pH-6.8, 100 mM KCl, 5 mM MgCl_2_, 5 mM EDTA, and 0.1% Triton X-100. This process was repeated twice. Further, the remaining pellet was washed in buffer (pH-6.8, 5 mM EDTA, and 100 mM KCl) and then centrifuged at 2500× *g* for 10 min. This washing step was repeated once more. The obtained myofibrillar pellet was then re-suspended in a buffer solution (5 mM tris-hydroxymethyl aminomethane and 150 mM KCl, pH-7.4). The supernatant was used to calculate the amount of myofibrillar protein. The concentrations of both total protein and myofibrillar protein were assessed using a Lowry assay [39]. Bovine serum albumin (BSA) was used as the standard.

### 2.7. Assessment of Oxidative Stress Markers and Antioxidative Status

To conduct the oxidative stress and antioxidant assay, 100 mg of the gastrocnemius (GN) muscle was homogenized in 1 mL of cold phosphate-buffered saline (pH 7.4). The homogenate was subsequently used for all biochemical estimations.

#### 2.7.1. Estimation of Lipid Peroxidation by Malondialdehyde

The measurement of malondialdehyde (MDA) content by thiobarbituric acid (TBA) directly determines the non-enzymatic oxidative state for lipid peroxidation [40]. In brief, 500 µL of 30% trichloroacetic acid (TCA), 500 µL of 0.8% TBA, and 1 mL of homogenized GN muscles in phosphate-buffered saline were mixed and incubated for 10 min at room temperature. The resulting reaction mixture was then heated at 80 °C for 30 min. After the mixture was cooled, it was centrifuged at 5000× *g* for 15 min, and the absorbance of the resulting supernatant was measured at 540 nm. The results are expressed in nM/mg protein.

#### 2.7.2. Estimation of Protein Carbonyl Content

The protein carbonyl content (PC) is determined based on the reaction of 2,4-Dinitrophenylhydrazine (DNPH) and serves as an indicator of oxidative stress [41]. Briefly, 500 µL of 10% TCA and 150 µL of GN muscle homogenate were mixed and incubated at room temperature for ten minutes. After centrifuging the reaction mixture at 13,000× *g* for 2 min, the supernatant was discarded, and the pellet was collected. Next, the pellet was suspended in 250 µL of 0.2% DNPH and incubated for 30 min. After incubation, 50 µL of 100% TCA was added to the mixture and centrifuged at 13,000× *g* for 5 min. The supernatant was then discarded, and the remaining cell pellets were 500 µL of washed with 1:1 *v*/*v* ethanol: ethyl acetate solution. The pellets were further dissolved in 6 M guanidine HCl and vortexed. Subsequently, the absorbance of the supernatant was measured at 360 nm. The results are expressed in µM/mg protein.

#### 2.7.3. Estimation of Catalase Activity by H_2_O_2_ Decomposition

Catalase activity was directly estimated by monitoring the rate of H_2_O_2_ breakdown. In brief, 50 µL of GN muscle homogenates were mixed with 250 µL of 19 mM of H_2_O_2_. Next, immediately after the reaction, we measured the absorbance at 240 nm for 3 min. Catalase activity was calculated using an extinction value of 0.0719 mM^−1^cm^−1^. The results are expressed as the number of moles of H_2_O_2_ that were broken down/min/mg of protein in the sample.

#### 2.7.4. Estimation of Reduced Glutathione Activity by Ellman’s Reagent

The amount of glutathione (GSH) was determined by reducing 5,5’-dithiobis-(2-nitrobenzoic acid) (also known as DTNB or Ellman’s reagent) by the thiol group of GSH, which resulted in a yellow-colored GS-TNB complex [42]. In brief, 250 µL of 50% TCA and 120 µL GN muscles homogenate was incubated for 10 min at room temperature. After centrifugation at 5000× *g* for 10 min, the precipitate was removed. In a reaction mixture comprising 3 µL of 0.6 mM DTNB and 130 µL of 0.2 M sodium phosphate-buffered (pH 8), free -SH groups in the supernatant were measured (pH 8.0). The absorbance was determined at 405 nm, and the results were expressed in nanomoles/mg of protein.

#### 2.7.5. Estimation of Superoxide Dismutase Activity by Pyrogallol Activity

Superoxide dismutase (SOD) activity was determined by inhibiting the reduction in pyrogallol activity by SOD present in the sample [43]. In brief, 2.9 mL of 0.5 M tris buffer (pH 8.0) having tris HCl (50 mM), EDTA (1 mM) was mixed with 100 µL of GN muscles homogenate. After that, incubation for 5 min was carried out at room temperature. A total of 25 µL of 2.6 mM pyrogallol was then added to stop the reaction. The change in absorbance after the addition of pyrogallol for 3 min was measured at 420 nm. SOD activity was evaluated in millimoles of reduced pyrogallol/min/mg of protein.

### 2.8. Estimation of Cellular Toxicity by Histological Analysis

The gastrocnemius (GN) muscles were preserved by fixing them in 10% formalin. Next, the muscles were subjected to a series of treatments involving graded alcohol and xylene, followed by embedding in paraffin wax. The sections were cut with a thickness of 5 µm using a Thermo HM325 rotary microtome and were taken on pre-coated slides. Then, the sections were dewaxed in xylene for 2–5 min and processed according to the previous protocol [44]. The muscle sections were stained with Hematoxylin and Eosin (H&E) and subsequently processed and mounted with DPX. Furthermore, the slides were observed at 20× magnification by using an Olympus Microscope (BX53, Hamburg, Germany). The cross-sectional area (CSA) and Feret’s diameter of the muscle fibers were analyzed by ImageJ software (NIH, Bethesda, MD, USA) [44]. A total of 15–20 tissue sections were utilized for analysis, with 3 slides per group.

### 2.9. Estimation of Serum Testosterone, GDF-8, Inflammatory Markers, and Lipid Markers

Serum levels of testosterone, GDF-8, IL-2, IL-6, IL-1β, creatine kinase, total cholesterol (TC), low-density lipoprotein (LDL), high-density lipoprotein (HDL), and triglycerides (TG) were measured using the procedure provided by the manufacturer. The very-low-density lipoprotein (VLDL) lipid level was determined using Friedewald’s formula as follows:

VLDL (mg/dL) = TC − HDL − LD

### 2.10. ^1^H NMR-Based GN Muscle Metabolomics Profiling

#### 2.10.1. Sample Preparation

In total, 50 mg GN muscle was homogenized in 500 µL of ice-cold normal saline. Homogenates were vortexed and sonicated for 30 s. After that, GN muscle homogenates were centrifuged at 16,278× *g* for 5 min at 4 °C. After homogenization, the supernatant was collected, and from this, 250 µL of supernatant was mixed with 250 µL of 100% deuterium oxide (D_2_O). Following this step, 450 µL of this mixture was put in 5 mm NMR tubes (Wilmad Glass, Vineland, NJ, USA). A sealed capillary tube containing sodium salt of 3-trimethylsilyl-(2,2,3,3-d4)-propionic acid (1.0 mM) (TSP) dissolved in D_2_O as a co-solvent was used as an internal standard reference and inserted in the NMR tubes [45].

#### 2.10.2. NMR Measurements

A Bruker 800 MHz NMR spectrometer (AVANCE-III, equipped with a cryoprobe) was used for NMR experiments at 298 K. The metabolic profiles of the muscles were determined using one-dimensional (1D) ^1^H CPMG (Carr–Purcell–Meiboom–Gill) pulse sequence NMR. The tests were performed on all muscle samples with pre-saturation of the water signal during a recycle delay (RD) of 5 s using the Bruker standard library pulse program “cpmgpr1d”. The other acquisition parameters were as follows: the width of the ^1^H spectral sweep was 12 ppm, the number of transients was 128, and the T2 filtering time (to suppress the broad signals of higher molecular weight macromolecules, including proteins) was obtained with an echo time of 200 s repeated 300 times, which resulted in a total effective echo time of 60 ms. All NMR spectra were processed using the Bruker NMR data processing program Topspin (v2.1), employing a typical Fourier transformation (FT) technique, as well as manual phase and baseline correction. Before the FFT was performed, each FID was zero-filled to a total of 65,536 data points and then multiplied by an exponential line-broadening function operating at 0.3 Hz.

#### 2.10.3. Spectral Assignment and Concentration Profiling

Using the Chenomx NMR suite’s 800 MHz compound spectral database library with pH set at 7.2 for all samples (Chenomx Inc., Edmonton, AB, Canada), various peaks in the ^1^D ^1^H CPMG NMR spectra were identified and annotated for different muscle tissue metabolites. This was performed in conjunction with 2D homonuclear ^1^H-^1^H TOCSY and heteronuclear ^1^H-^13^C HSQC NMR [46]. The metabolic assignments were further confirmed using publicly accessible databases (such as HMBD: http://www.hmdb.ca (accessed on 21 October 2022) and BMRB: www.bmrb.wisc.edu/metabolomics (accessed on 15 September 2022) and and the NMR assignments of metabolites published in several previous metabolomics studies [47,48,49,50]. All CPMG pulse NMR spectra that were collected were visually inspected to determine whether they were acceptable. Additional analyses were performed using the NMR suite of the commercial software CHENOMX (Chenomx Inc., Edmonton, AB, Canada). First, all of the NMR spectra were baseline corrected and calibrated internally to ^1^H NMR peak of formate (at *δ* = 8.43 ppm and with the concentration set to 0.01 mM). Next, concentration profiling of the 40 muscle tissue metabolites was carried out following the procedure described in the previous section [51]. Then, 3-hydroxy-butyrate (3HB), acetone, alanine, betaine, acetate, choline, citrate, dimethyl sulfone (DMS), creatine, dimethylamine (DMA), glutamine, glucose, glutamate, glycine, glycerol, isoleucine, isobutyrate (IsoB), leucine, lactate, phenylalanine, methanol, pyruvate, succinate, proline, serine, threonine, valine, tyrosine, histidine, and myoinositol. Following this, the metabolic profiles were utilized to estimate five key metabolic ratios, including the phenylalanine-to-tyrosine ratio (PTR), histidine-to-tyrosine ratio (HTR), and glutamate-to-glutamine ratio (EQR), as discussed in the preceding section [52,53].

#### 2.10.4. Multivariate Data Analysis

The resultant metabolic concentrations and ratio values were then transferred to the MS Office Excel program and transformed into a comma-separated values (CSV) text format file, which was then utilized for multivariate data analysis in MetaboAnalyst 4.0., which is an open-access web-based metabolomics data processing tool [54,55]. Principal component analysis, often known as PCA, was applied to locate data outliers and offer a concise picture of the trending grouping of the data set. Subsequently, supervised Partial Least Squares Discriminant Analysis (PLS-DA) was applied to discover group separations and locate the discriminating metabolites that accounted for group separations. A 10-fold cross-validation approach was used to prevent overfitting of the PLS-DA model. For GN muscle metabolic profiling, NMR spectra acquired from the muscle tissue were processed using the PROFILER-Module of CHENOMX. Following this, the quantities of the selected metabolites were determined for each of the three sets of muscle tissue samples. PLS-DA analysis was used to evaluate the quantitative muscle metabolic profiles of the control, salbutamol, HFD/STZ, and HFD/STZ + salbutamol groups. Permutation analysis was performed to cross-validate the PLS-DA models a hundred times, and the resultant goodness-of-fit parameter R^2^ and goodness-of-prediction parameter Q2 were utilized to evaluate the characteristics of the PLS-DA models. The VIP score, also known as the variable significance on projection score, must have a value larger than 1.0 to be employed in the PLS-DA model’s process of identifying the metabolites responsible for discrimination. The metabolite concentration profile data matrix was then exposed to a random forest (RF) classification model (a supervised machine learning method), and discriminatory metabolic profiles were cross-validated using a Mean Decrease Accuracy (MDA) score plot [56,57]. In summary, MDA reflects how much accuracy the model loses when each variable is removed from the RF classification model when comparing the study groups. The greater the loss of accuracy, the more critical the variable is for effective categorization. The statistical analysis module of MetaboAnalyst (https://www.metaboanalyst.ca (accessed on 21 October 2022)) was used to analyze the RF classification model. Analysis of variance (ANOVA) with multiple group comparisons was used to assess the statistical significance of the discriminating factors. The benchmark for statistical significance was a significance level of 0.05 or a *p*-value of 0.05. Chenomx NMR Suite v8.1 was used to quantitatively estimate the key metabolites.

### 2.11. Statistical Analysis

The data were analyzed using GraphPad Prism software (version 8.01). All the values were expressed as the mean ± standard deviation (SD). Statistical analysis was performed on the data from the four groups using a one-way analysis of variance (ANOVA), followed by Tukey’s multiple comparisons *post hoc* test. The differences were considered statistically significant at *p* < 0.05.

## 3. Results

### 3.1. Effect of Salbutamol on Blood Glucose Levels, Body Weight, and GN Muscle Weight in HFD/STZ-Induced Diabetic Rats

There was no change in blood glucose levels between the HFD/STZ and HFD/STZ + salbutamol groups (Table 1). These results suggest that salbutamol treatment did not have a significant effect on blood glucose levels. Similarly, the fasting insulin levels were 399.75 ± 9.35 pg/mL in diabetic rats when compared to control (860.58 ± 11.58 pg/mL). SLB treatment for 4 weeks led to a significant increase in body weight as compared to the HFD/STZ group (*p* < 0.001). These results indicate that SLB treatment leads to a decrease in body weight in the HFD/STZ group. The finding also showed that SLB treatment significantly increased the GN weight as compared to the HFD/STZ group (*p* < 0.001) (Table 1). Findings indicate that SLB treatment increased the weight of the GN muscle in HFD/STZ rats. These results led us to further investigate body composition, specifically the measurements of lean mass and fat mass.

### 3.2. Effect of Salbutamol on Body Composition in HFD/STZ-Induced Diabetic Rats

**Figure 1:**
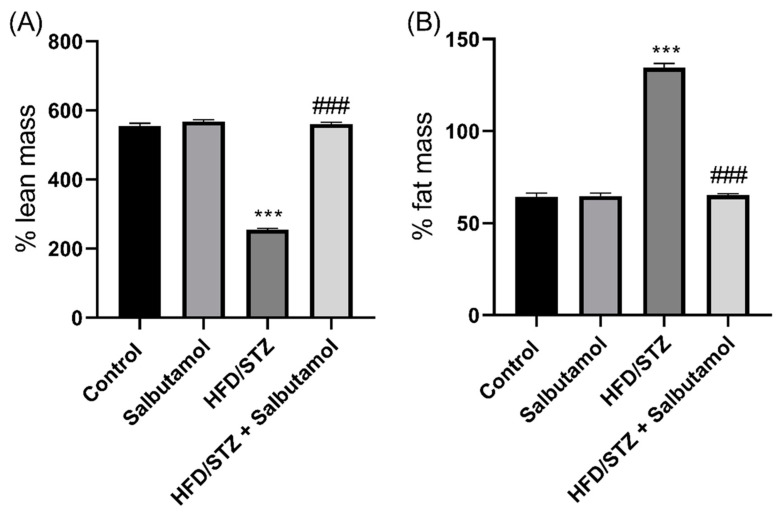
Effect of salbutamol on the lean muscle mass in HFD/STZ -induced diabetic rats. (**A**) Lean mass (%) and (**B**) fat mass (%). Data were represented as mean ± SD (*n* = 6). Statistical significance was determined using one-way ANOVA with Tukey’s multiple comparisons post hoc test. Bar graphs depict *** p < 0.001 vs. control and *^###^ p <* 0.001 vs. HFD/STZ.

The results demonstrated that SLB treatment significantly increased the percentage of lean mass compared to the HFD/STZ group (*p* < 0.001) (Figure 1A). This suggests that SLB treatment led to an increase in lean tissue or muscle mass in HFD-induced diabetic rats. The SLB treatment group exhibited a reduced percentage of fat mass compared to the HFD/STZ group (*p* < 0.001) (Figure 1B). This indicates that SLB treatment was associated with a decrease in fat tissue in HFD/STZ-induced diabetic rats. These findings suggest that SLB may have a positive effect on body composition by promoting an increase in lean mass and reduced fat mass in HFD/STZ-induced diabetic rats. The reduced percentage of fat mass and the greater percentage of lean mass in the SLB treatment group imply that SLB could potentially ameliorate muscle atrophy associated with the HFD/STZ-induced diabetic condition. These results further evaluate muscle strength and coordination in HFD/STZ-induced diabetic rats.

### 3.3. Effect of Salbutamol on Muscle Strength and Motor Coordination in HFD/STZ-Induced Diabetic Rats

**Figure 2:**
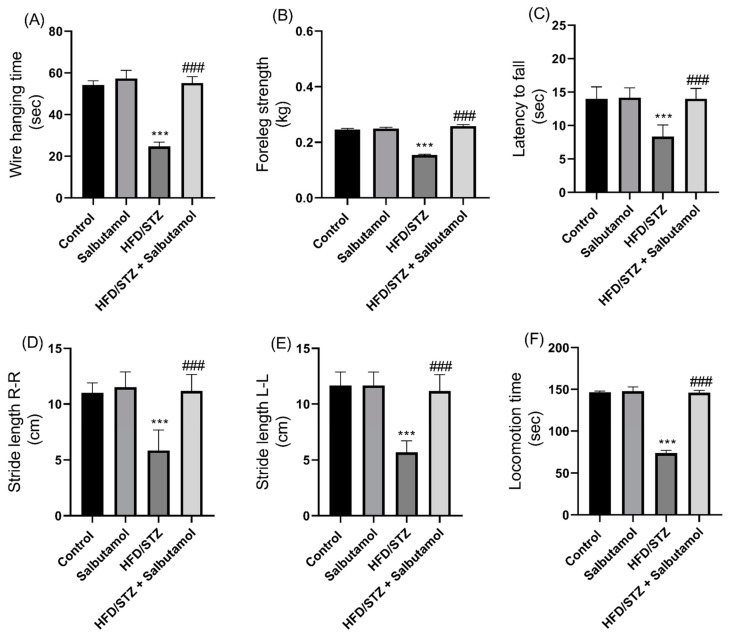
Effect of salbutamol on muscle strength, function and coordination in HFD/STZ-induced diabetic rats. (**A**) Hanging time (s), (**B**) Foreleg grip strength (kg), (**C**) Latency to fall (s), (**D**) Stride length (cm) R-R, (**E**) Stride length (cm) L-L, and (**F**) Locomotion time (s). Data were represented as mean ± SD (*n* = 6). Statistical significance was determined using one-way ANOVA with Tukey’s multiple comparisons post hoc test. Bar graphs depict *** p < 0.001 vs. control and *^###^ p <* 0.001 vs. HFD/STZ.

Next, diabetic rats exhibited a significant decrease in grip strength and wire-hanging ability, indicating compromised muscle strength compared to the control group. However, the SLB treatment improved grip strength and wire-hanging performance compared to the control group (*p* < 0.001) (Figure 2A,B). SLB treatment significantly increased muscle coordination and balance. This was demonstrated by increased latency to fall (*p* < 0.001) (Figure 2C) and longer stride lengths (right-to-right and left-to-left) (*p* < 0.001) (Figure 2D,E) in diabetic rats. SLB treatment also resulted in increased locomotion time in diabetic rats (*p* < 0.001) (Figure 2F). This suggests an improvement in motor coordination and balance due to SLB intervention. Overall, the results indicate that SLB treatment effectively improved muscle strength, coordination, function, and balance in HFD/STZ-induced diabetic rats. The improvements observed in grip strength, wire-hanging ability, latency time, stride lengths, and locomotion time suggest that SLB can improve muscle strength and enhance motor coordination.

### 3.4. Effect of Salbutamol on Total and Myofibrillar Protein Concentration in HFD/STZ-Induced Diabetic Rats

**Figure 3:**
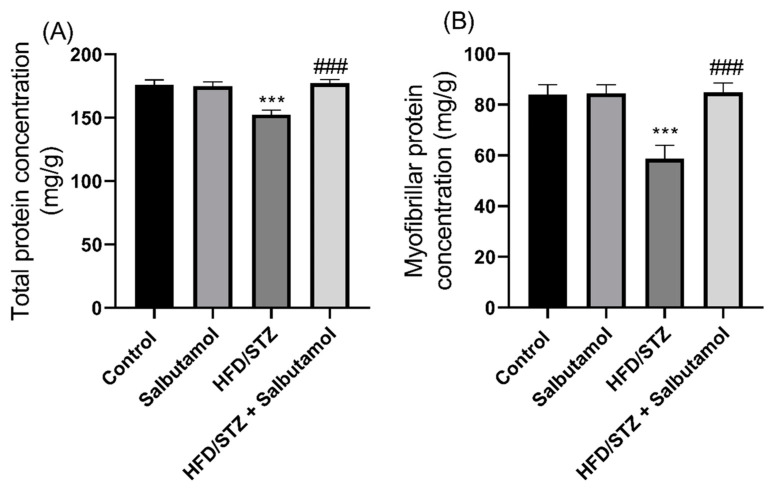
Effect of salbutamol on the protein concentration of GN muscle in HFD/STZ-induced diabetic rats. (**A**) Total protein concentration (mg/g) and (**B**) myofibrillar protein concentration (mg/g). Data were represented as mean ± SD (*n* = 6). Statistical significance was determined using one-way ANOVA with Tukey’s multiple comparisons *post hoc* test. Bar graphs depict *** p < 0.001 vs. control and *^###^ p <* 0.001 vs. HFD/STZ.

Next, the total protein content in the GN muscles was significantly decreased in the HFD/STZ group compared to the control group (*p* < 0.001). SLB treatment was able to restore the total protein content compared to the HFD/STZ group (Figure 3B). Similarly, the myofibrillar protein content was found to be significantly decreased in the HFD/STZ group compared to the control group (*p* < 0.001). However, SLB treatment restored the myofibrillar protein content (Figure 3A). These results suggest that the HFD/STZ-induced diabetic condition led to a reduction in both total protein contents and myofibrillar protein in the GN muscles. However, SLB treatment significantly increased the protein contents, indicating its potential to counteract the negative effects of the HFD/STZ-induced diabetic condition on skeletal muscle protein.

### 3.5. Effect of Salbutamol on Oxidative Stress and Antioxidant Status in HFD/STZ-Induced Diabetic Rats

**Figure 4:**
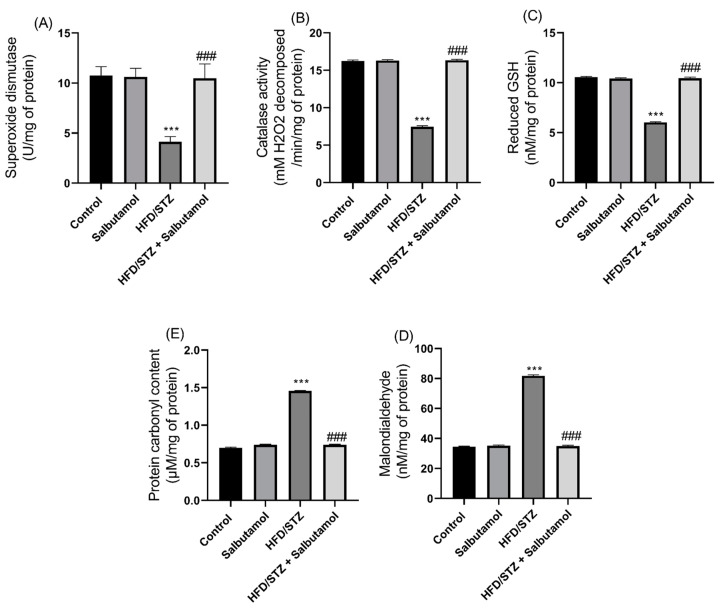
Effect of salbutamol on antioxidant status and oxidative stress markers of GN muscle in HFD/STZ-induced diabetic rats. (**A**) Superoxide dismutase (U/mg of protein), (**B**) catalase activity (U/mg), (**C**) reduced GSH (nM/mg of protein), (**D**) MDA (nM/mg of protein), and (**E**) protein carbonyl content (µmoles/mg of protein). Data were represented as mean ± SD (*n* = 6). Statistical significance was determined using one-way ANOVA with Tukey’s multiple comparisons post hoc test. Bar graphs depict *** p < 0.001 vs. control and *^###^ p <* 0.001 vs. HFD/STZ.

Next, the levels of superoxide dismutase (SOD), catalase, and reduced glutathione (GSH) antioxidant enzymes were found to be significantly decreased in the HFD/STZ group compared to the control group (*p* < 0.001). However, SLB treatment restored the antioxidant status by increasing the levels of SOD, catalase, and GSH, similar to the control group (Figure 4A–C). The levels of malondialdehyde (MDA), which is a marker of lipid peroxidation, and protein carbonyl (PC), which is a marker of protein peroxidation, were significantly increased in the HFD/STZ group compared to the control group (*p* < 0.001). However, SLB treatment resulted in decreased levels of oxidative stress, as evidenced by reduced levels of MDA and PC (Figure 4D,E). These results indicate that the diabetic condition resulted in increased oxidative stress and decreased antioxidant status in the GN muscles. Furthermore, the treatment with SLB significantly increased the antioxidant status and reduced oxidative stress in the diabetic muscle.

### 3.6. Effect of Salbutamol on the Cellular Architecture of GN Muscle in HFD/STZ-Induced Diabetic Rats

**Figure 5:**
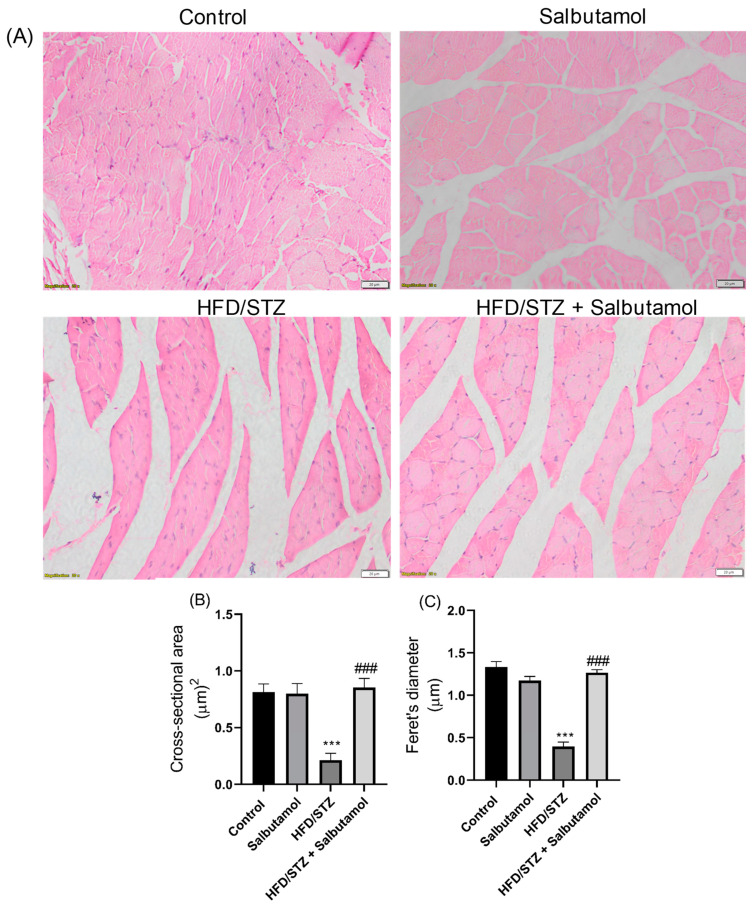
Effect of salbutamol on muscle architecture in HFD/STZ-induced diabetic rats (**A**) H&E-stained images of GN muscle, (**B**) cross-sectional area (µm)^2^, and (**C**) Feret’s diameter (µm). Data were represented as mean ± SD (*n* = 6). Statistical significance was determined using one-way ANOVA with Tukey’s multiple comparisons post hoc tests. Bar graphs depict *** p < 0.001 vs. control and *^###^ p <* 0.001 vs. HFD/STZ.

The GN muscles in the control group showed a regular and organized structure, indicating normal muscle morphology (Figure 5A). In diabetic rats, the GN muscles exhibited shrinkage and varying myofiber sizes with significant gaps when compared to the control group. This suggests muscle damage and disorganization associated with the HFD/STZ-induced diabetic condition (Figure 5A). SLB treatment restored the muscle architecture in the HFD/STZ group, indicating a reversal of the structural abnormalities induced by diabetes (Figure 5A). In addition, quantitative measurements of the cross-sectional area (CSA) and Feret’s diameter of myofibers were decreased in the HFD/STZ group compared to the control group and were performed using ImageJ software 1.44 (NIH, USA). The results demonstrated that salbutamol treatment significantly increased the CSA and Feret’s diameter of myofibers in diabetic rats compared to the control group (*p* < 0.001). This indicates an improvement in myofiber size and morphology following salbutamol intervention (Figure 5B,C). These findings suggest that the intervention of salbutamol effectively improved the cellular changes and structural abnormalities in the GN muscle associated with HFD/STZ-induced diabetes. The restoration of muscle architecture, as evidenced by the increased cross-sectional area (CSA) and Feret’s diameter of myofibers, indicates the potential of salbutamol in ameliorating muscle cellular architecture in diabetic conditions.

### 3.7. Effect of Salbutamol on Serum Level of Creatine Kinase, GDF-8, Testosterone, and Pro-Inflammatory Markers in HFD/STZ-Induced Diabetic Rats

**Figure 6:**
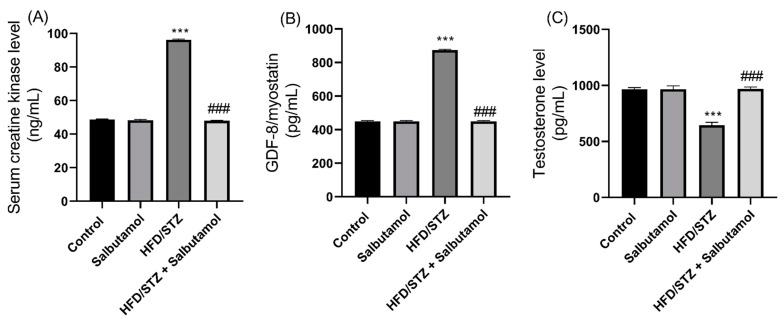
Effect of salbutamol on muscle damage markers in HFD/STZ-induced diabetic rats. (**A**) Serum creatine kinase (ng/mL), (**B**) GDF-8 level (pg/mL), and (**C**) Testosterone level (pg/mL). Data were represented as mean ± SD (*n* = 6). Statistical significance was determined using one-way ANOVA with Tukey’s multiple comparisons post hoc test. Bar graphs depict *** p < 0.001 vs. control and *^###^ p <* 0.001 vs. HFD/STZ.

**Figure 7:**
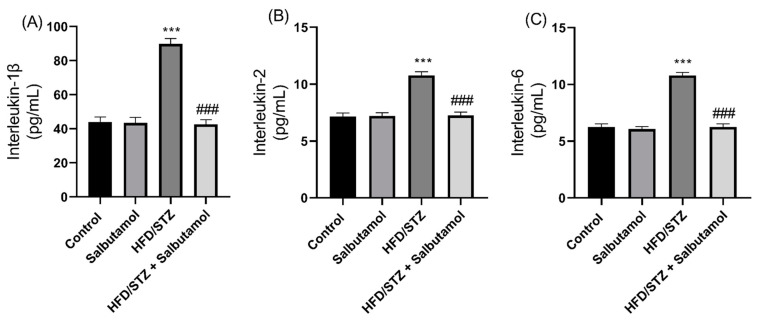
Effect of salbutamol on serum inflammatory markers in HFD/STZ-induced diabetic rats. (**A**) IL-1β (pg/mL), (**B**) IL-2 (pg/mL), and (**C**) IL-6 (pg/mL). Data were represented as mean ± SD (*n* = 6). Statistical significance was determined using one-way ANOVA with Tukey’s multiple comparisons post hoc test. Bar graphs depict *** p < 0.001 vs. control and *^###^ p <* 0.001 vs. HFD/STZ.

Next, the serum creatine kinase (CK) and growth differentiation factor 8 (GDF-8) muscle damage markers were found to be significantly elevated in HFD/STZ group compared to the control group (*p* < 0.001). However, SLB treatment significantly decreased serum CK and GDF-8 levels in the diabetic group (Figure 6A,B). These results suggest that the decreased levels or inhibition of myostatin and CK, in combination with SLB treatment, may enhance skeletal muscle mass. The serum testosterone level was increased in the SLB treatment group compared to the HFD/STZ group (*p* < 0.001). This increase in testosterone level was positively correlated with muscle mass, suggesting a potential role of testosterone in the modulation of muscle mass (Figure 6C). Also, the serum pro-inflammatory markers IL-1β, IL-2, and IL-6 were found to be significantly increased in the HFD/STZ group compared to the control group (*p* < 0.001). However, SLB intervention resulted in reduced levels of these pro-inflammatory cytokines (IL-1β, IL-2, and IL-6) (Figure 7A–C). This indicates that lowered pro-inflammatory cytokine levels may attenuate muscle atrophy. These results suggest that HFD/STZ-induced diabetic conditions lead to alterations in circulating markers associated with muscle mass, inflammation, and testosterone levels. SLB treatment appears to have a beneficial effect by restoring the levels of CK and GDF-8, positively influencing testosterone levels, and reducing pro-inflammatory cytokine levels. These findings highlight the potential of SLB in reducing muscle inflammation in diabetic rats.

### 3.8. Effect of Salbutamol on Serum Lipid Profile in HFD/STZ-Induced Diabetic Rats

**Figure 8:**
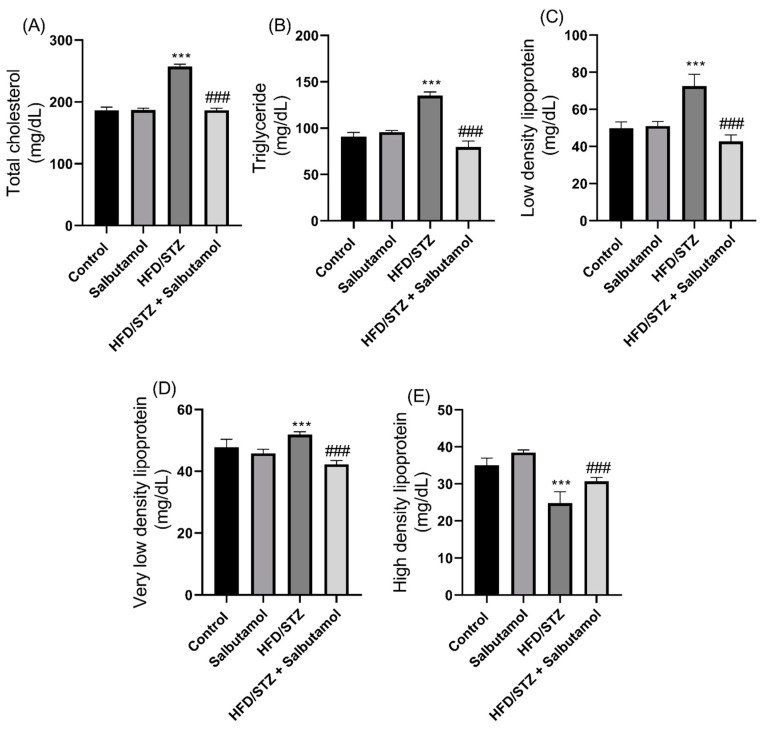
Effect of salbutamol on serum lipidemic profile in HFD/STZ-induced diabetic rats. (**A**) Total cholesterol (mg/dL), (**B**) Triglyceride (mg/dL), (**C**) HDL (mg/dL), (**D**) LDL (mg/dL), and (**E**) VLDL (mg/dL). Data were represented as mean ± SD (*n* = 6). Statistical significance was determined using one-way ANOVA with Tukey’s multiple comparisons post hoc test. *** p < 0.001 vs. control and *^###^ p <* 0.001 vs. HFD/STZ.

The HFD/STZ group exhibited significantly higher levels of total cholesterol (TC), triglyceride (TG), low-density lipoprotein (LDL), and very-low-density lipoprotein (VLDL) compared to the control group (*p* < 0.001) (Figure 8A–D). These findings indicate an elevation in circulating lipid levels associated with the diabetic condition. The HDL levels in the HFD/STZ group were significantly lower than those in the control group (*p* < 0.001) (Figure 8E). This indicates a reduction in the levels of protective HDL cholesterol in diabetic rats. SLB intervention significantly restored the serum lipid profiles, indicating a reversal of the lipid abnormalities induced by diabetes (Figure 8A–E). These results suggest that the diabetic condition leads to dysregulation of serum lipid profiles, characterized by elevated levels of TC, LDL, VLDL, and TG, as well as reduced levels of HDL. The administration of SLB appears to ameliorate these lipid abnormalities by restoring the lipid profile to levels compared to the control group. These findings suggest that excessive lipid levels may contribute to muscle damage, and the intervention with SLB helps in mitigating this effect.

### 3.9. Effect of Salbutamol on GN Muscle Metabolomics Using ^1^H NMR-Based Technique in HFD/STZ-Induced Diabetic Rats

**Figure 9:**
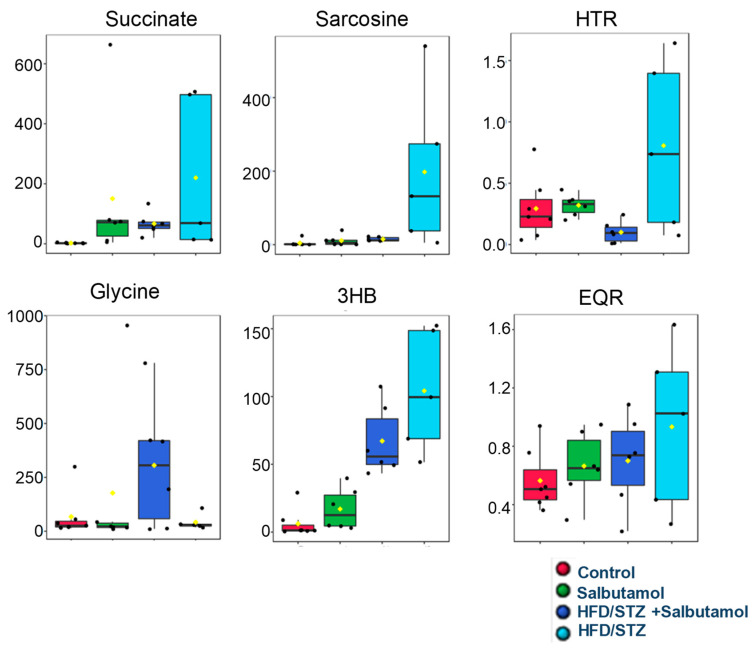
Representative box-cum-whisker plots showing quantitative variations of concentration pertinent GN muscle metabolites. The black round dots along the Y axis in the box plots denote the concentrations of metabolites, while the yellow rhombus denotes mean concentrations of the group. In the box plots, the boxes denote interquartile ranges, horizontal lines inside the box denote the median, and the bottom and top boundaries of boxes are 25th and 75th percentiles, respectively. Lower and upper whiskers are 5th and 95th percentiles, respectively. Key acronyms are HTR: histidine-to-tyrosine ratio 3-HB: 3-hydroxybutyrate; EQR: glutamate-to-glutamine ratio.

Figure S1 shows the typical 1D and ^1^H NMR spectra of rat GN muscle samples acquired from the control, salbutamol, HFD/STZ, and HFD/STZ + salbutamol groups. NMR peaks of the different metabolites were annotated. They mainly show the signals of metabolites, such as (a) amino acids, viz. alanine, glycine, glutamate, glutamine, π-methylhistidine, leucine, isoleucine, phenylalanine, methionine, sarcosine, proline, threonine, serine, valine, and tyrosine; (b) energy metabolites, viz. acetate, creatine, fumarate, formate, glycerol, lactate, pyruvate, and succinate; (c) lipoproteins (VLDL and LDL); (d) ketone body content, viz. acetone, betaine, 3-hydroxybutyrate; and (e) additional metabolites were also estimated in the subsequent combinations or ratios, viz. phenylalanine-to-tyrosine ratio (PTR), histidine-to-tyrosine ratio (HTR), and glutamate-to-glutamine ratio (EQR). Next, to investigate the effect of salbutamol on metabolites in diabetic rats, the GN muscle metabolic profile was obtained using 1D ^1^H NMR spectroscopy. This profile was then subjected to multivariate statistical analysis to determine the metabolic patterns that were altered. This was carried out with the help of MetaboAnalyst (v4.0, a free web-based software [58]. The 3D score plot obtained from PLS-DA analysis showed a distinct separation among the four groups, demonstrating a substantial metabolic difference between the HFD/STZ and HFD/STZ + salbutamol groups as opposed to the control group and the salbutamol group by itself. (Figure S2A). In addition, compared with the standard HFD/STZ group, there was a discernible tendency toward clustering treatment groups (HFD/STZ + salbutamol) and shifting toward the control group (Figure S2A). The PLS-DA model validation parameters (R2 > 0.57 and Q2 > 0.38) and predictive capability (Q2) were significantly high, suggesting a significant metabolic variation between the study groups (Figure S2B). The metabolic features of discriminatory relevance were first identified using the PLS-DA model based on variable importance in projection (VIP) score values > 1.0 (Figure S2C). Statistical significance was evaluated using ANOVA. We also performed machine learning random forest (RF) classification analysis (RFA) to confirm the discriminatory potential of metabolic profiles for classifying the data. The variables are presented from descending importance in the mean decrease accuracy (MDA) score plot derived from the RF clustering approach. Integrative analysis (based on VIP and MDA score plots and ANOVA statistics) identified several metabolic entities with discriminatory potential. It could predict the therapeutic response to salbutamol, as shown in Figures S2C,D. Compared to control rats, GN muscle levels of 3-hydroxybutyrate, sarcosine, succinate, HTR, PTR, and EQR were elevated, and creatine and glycine levels were decreased in the HFD/STZ group (Figure S2B,D). Quantitative variations in these discriminatory features are shown through box-cum-whisker plots (Figure 9). As evident from the results, several GN muscle metabolites showed a metabolic reprogramming trend (Figure 9), suggesting that salbutamol could potentially alleviate metabolic alterations in diabetic rat muscle.

### 3.10. Disturbed Interlinking Metabolic Pathways in Diabetes-Induced Skeletal Muscle Atrophy

**Figure 10:**
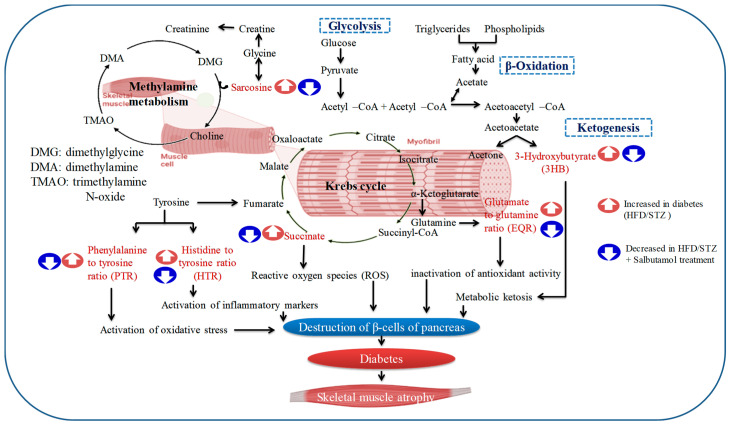
The figure illustrates the involvement of specific enzymes and their impact on interconnected pathways, offering valuable insights into understanding the intricacies of muscle metabolism. The representative altered key metabolites in the figure depict interlinked pathways, including glycolysis, the tricarboxylic acid (TCA) cycle, and methylamine metabolism. The blue color signifies the restored metabolites due to salbutamol intervention, demonstrating the ameliorating effect of salbutamol on diabetic skeletal muscle. In contrast, the dark red color indicates the altered status of metabolites in the diabetic condition, indicating significant increases and imbalances within different pathways of skeletal muscle.

After analyzing significant muscle metabolites (sarcosine, PTR, EQR, HTR, succinate, and 3-hydroxybutyrate) in HFD/STZ and HFD/STZ + salbutamol groups, we investigated how those metabolites are involved and utilized in glycolysis, TCA cycle, and other metabolic pathways. These pathways included the tricarboxylic acid (TCA) cycle (succinate), ketogenesis (3-hydroxybutyrate), histidine metabolism (histidine), glycine metabolism (sarcosine), and other metabolic pathways (PTR, HTR, and EQR) in Figure 10 and are detailed in the Section 4.

## 4. Discussion

In the present study, salbutamol significantly improved muscle mass, grip strength, antioxidant levels, and muscle architecture in HFD/STZ-induced diabetic rats. Furthermore, muscle metabolomics analysis showed that salbutamol significantly restored the altered metabolites such as sarcosine, HTR, PTR, EQR, succinate, and 3-hydroxybutyrate in diabetic rats. Taken together, these findings showed that salbutamol attenuated skeletal muscle atrophy in HFD/STZ-induced diabetic rats. The results showed that salbutamol significantly increased body weight and gastrocnemius (GN) muscle weight but did not alter blood glucose levels in HFD/STZ-induced diabetic rats. Emery et al. showed that 16 days of treatment with β2 agonists, viz. clenbuterol and fenoterol, increased the body weight and GN muscle mass as compared to the control rat [59]. A low dose of clenbuterol improved glucose homeostasis in insulin-resistant rats. This was most likely mediated by increasing glucose absorption in skeletal muscle, which increased insulin sensitivity. A finding showed that salbutamol increased the GN muscle weight and protein content in both young and senescent rats [19]. Clinical studies in human subjects demonstrate that salbutamol treatment increased lean body mass, muscle strength, and endurance [20]. Our findings also showed that salbutamol significantly increased the lean mass and grip strength in diabetic rats. These findings indicate that salbutamol has the potential to increase muscle mass. Furthermore, protein accretion is linked to skeletal muscle growth, and muscle protein pools primarily contain myofibril, mitochondrial, and sarcoplasmic proteins [20]. Our results showed that salbutamol had a higher myofibrillar protein content in GN muscle as compared to diabetic rats. Recent findings have demonstrated that the β2-agonist salbutamol promotes the transition of the muscle fiber isoform from MHC-I to MHCIIa [21]. Moreover, salbutamol intervention in resistance exercise increased the myofibrillar protein fractionation rate and turnover in young men [20]. Next, we checked the levels of testosterone and muscle damage markers, viz. creatine kinase and myostatin. Intriguingly, treatment with salbutamol increased testosterone levels in diabetic rats, which is consistent with the finding that salbutamol considerably increased plasma testosterone levels in athletic men following submaximal exercise [60]. Myostatin (also known as GDF-8) is a negative regulator that affects skeletal muscle growth [61]. Our results showed that salbutamol significantly decreased myostatin levels in diabetic rats. The results were also in line with findings where type 2 diabetic subjects showed increased mRNA expression of muscle myostatin linked to metabolism and systemic inflammation [62]. Contrary to the results presented here, salbutamol was shown to increase myostatin mRNA levels; however, this effect could be neutralized by the concomitant downregulation of activinRIIB, which is associated with resistance training [20]. Our results showed that salbutamol significantly decreased serum creatine kinase (CK) levels in diabetic rats. Because muscle has a large phosphocreatine reserve, changes in CK levels are associated with muscle injury and inflammation. Increased activity in the muscle implies muscular damage and CK leakage from the muscles. Earlier findings showed that creatine kinase levels were elevated in diabetic rats [63]. Thus, elevated serum CK levels observed in exercise-induced muscle damage can lead to the activation of pro-inflammatory markers. This activation can occur due to the destabilization of the cell and membrane, resulting in the infiltration of leukocytes during the process of repair. Next, we assessed the levels of serum inflammatory markers, including IL-2, IL-6, and IL-1β, and observed a significant increase in these markers in diabetic rats. However, our findings indicated that salbutamol treatment significantly restored these levels, suggesting that salbutamol possesses anti-inflammatory properties. Further, our results demonstrated that salbutamol significantly restored the levels of superoxide dismutase, catalase, and glutathione in the GN muscle of diabetic rats. These enzymes and molecules act as antioxidants, and their restoration by salbutamol suggests its potential role in mitigating oxidative stress in diabetic conditions.

Furthermore, GN muscle oxidative stress (MDA and protein carbonyl content) levels were significantly decreased by salbutamol treatment in diabetic rats. Previous studies have shown that salbutamol exerts significant antioxidative effects in rat models [64]. Moreover, our results showed that salbutamol treatment significantly increased the muscle fiber size and cross-sectional area in diabetic muscle. These findings demonstrate that salbutamol can attenuate muscle cellular architecture, highlighting its potential anti-atrophy properties in diabetic-induced skeletal muscle wasting.

Previous findings have demonstrated that the β-2 agonist formoterol significantly increases muscle fiber size, area, and contractile performance in skeletal muscles [17]. Taken together, our findings support the notion that beta-2 agonists could be effective interventions against muscle wasting disorders.

Next, we carried the ^1^H NMR muscle metabolomics to investigate the effects of salbutamol on diabetes-induced alterations in muscle metabolites. Findings showed that amino acids metabolites (e.g., glycine, histidine, tyrosine, and sarcosine), energy metabolites (e.g., choline, acetate, creatine, lactate, and succinate), and ketone bodies (e.g., betaine, 3-hydroxybutyrate, and isobutyrate) were significantly altered in diabetic skeletal muscles. Furthermore, metabolites levels of succinate, 3-hydroxybutyrate (3-HB), and the ratios of histidine-to-tyrosine (HTR), phenylalanine-to-tyrosine (PTR), and glutamate-to-glutamine ratio (EQR), were elevated in the HFD/STZ-induced diabetic group, and salbutamol treatment restored these levels (Figure 10). Succinate is primarily recognized as a metabolite that serves as an intermediate in the tricarboxylic acid (TCA) cycle and plays a vital role in mitochondrial metabolism and ATP generation. However, recent studies have revealed that succinate has broader implications beyond being a substrate for succinate dehydrogenase and the respiratory chain [65]. Our findings indicated a significant increase in succinate levels in diabetic muscle while salbutamol treatment restored the same. Changes in the altered levels of succinate (an intermediate of the TCA cycle) may affect energy balance and muscle insulin sensitivity [66]. Moreover, recent in vitro and in vivo studies have demonstrated that succinate supplementation can disrupt skeletal muscle homeostasis and impair muscle regeneration [65]. These findings suggest that defects in the TCA cycle are commonly observed in wasting skeletal muscle, and diabetic muscle is not an exception. Furthermore, previous metabolomics and transcriptomic studies have shown that the loss of succinate dehydrogenase (SDH) results in an excessive accumulation of succinate [67], which is consistent with the findings of our study. These studies have also revealed that SDH deficiency leads to the inappropriate activation of the mTORC1 pathway in β-cells, subsequently leading to mitochondrial dysfunction [67]. Overall, succinate appears to have multifaceted roles beyond its traditional function in the TCA cycle. Its dysregulation may contribute to skeletal muscle dysfunction and impaired regeneration observed in various pathological conditions, including diabetes [65]. Furthermore, intervention with salbutamol resulted in the restoration of the altered skeletal muscle metabolites. Our findings demonstrated a significant increase in 3-HB levels in the skeletal muscle of diabetic rats compared to the control group. Further, increased production of ketone bodies, such as β-hydroxybutyrate and acetoacetate, results in ketonemia. Moreover, the findings revealed that α-hydroxybutyrate, an organic acid derived from α-ketobutyrate, is a potential biomarker of insulin sensitivity in individuals with normal glucose tolerance. These findings suggest that impaired glucose metabolism and disrupted insulin regulation in diabetes indicate disturbances in fatty acid oxidation [68,69]. An elevated level of circulatory PTR (for oxidative markers) and HTR (for inflammatory markers) indicates that disease-induced oxidative stress and inflammation, and hyper-activation of the immune system led to predicated disease [53,70]. PTR and HTR assess the body’s capacity to convert phenylalanine to tyrosine and the histidine-to-tyrosine ratio. The conversion enzyme requires cofactors, such as tetrahydrobiopterin (BH4), niacin (B3), and iron. Increasing the muscle PTR ratio may aid in diagnosing inflammatory disease and a person’s catabolic stage [71]. To preserve physiological homeostasis and fulfill the energy demands of muscle cells, it is necessary to increase their dependence on additional energy sources [72]. As a direct result, several gluconeogenic amino acids, including glutamine and glutamate, were present at lower concentrations in the diabetic group. The consumption of glutamine is directly associated with the suppression of inflammatory reactions in skeletal muscle [73]. In addition, extracellular glutamine concentration can control the production of the adaptor protein GRB10 and has a direct impact on the muscle’s inflammatory response [73]. Next, sarcosine, chemically known as N-methyl glycine, is an intermediate in glycine biosynthesis and degradation and a glycine transporter inhibitor. However, sarcosine levels are governed by sarcosine dehydrogenase, an enzyme that converts sarcosine to glycine, and dimethylglycine dehydrogenase, which produces sarcosine from dimethylglycine. Owing to their capacity to regulate sarcosine levels in myonuclei cells, these enzymes may be essential for controlling sarcomere protein degradation [74]. Our ^1^H-NMR metabolomics analysis identified an intriguing and less-studied metabolite called sarcosine. It revealed a significant increase in sarcosine levels in diabetic muscle compared to the control group. Only one study has shown elevated sarcosine levels in rats exposed to a high-fructose and high-fat diet, utilizing LC/TOF-MS urine metabolomics analysis [75]. Interestingly, sarcosine has drawn attention as a potential biomarker for aggressive and metastatic prostate cancer, as elevated levels have been observed in tumors associated with this condition [76]. Sarcosine has been shown to activate autophagy in cultured cells and boosts autophagic flux in vivo, suggesting that it may play a role in the induction of autophagy caused by dietary constraints [77]. Additionally, a recent study published in the ‘Lancet’ found higher sarcosine levels in individuals who exhibited resistance to dietary changes following exercise training. This study involved muscle metabolomics combined with MS/MS analysis of plasma amino acids [78]. Taken together, these findings suggest that sarcosine may play a role in metabolic diseases and provide new evidence highlighting its significance in diabetic skeletal muscles. However, further research is necessary to fully elucidate the role of sarcosine in these conditions and explore its potential as a diagnostic or therapeutic target.

## 5. Conclusions

In conclusion, the findings of this study suggest that salbutamol has the potential to improve skeletal muscle atrophy in diabetic rats. Our results demonstrated that salbutamol significantly increased grip strength and lean muscle mass in diabetic rats. Also, salbutamol treatment results in increased levels of antioxidants in the muscles and reduced muscle atrophy and inflammatory markers, and restored muscle damage biomarkers that indicate its potential to reduce muscle inflammation and oxidative stress. Furthermore, the GN muscle metabolomics markers identified in this study could serve as valuable prognostic markers for diabetic skeletal muscle atrophy. Overall, these findings highlight the potential of salbutamol as a therapeutic intervention for managing skeletal muscle atrophy associated with diabetes.

## 6. Limitation of the Study

In the present study, our focus was solely on investigating the effects of salbutamol on lean mass, oxidative stress, inflammatory markers, and muscle metabolomics of gastrocnemius muscles in HFD/STZ-induced type 2 diabetic rats. The main limitation of this study is that we did not assess the protein expressions of muscle damage markers or investigate the specific mechanism by which salbutamol balances protein synthesis and degradation. Further, more preclinical studies and detailed molecular mechanisms will provide a comprehensive understanding of how salbutamol reduces muscle atrophy in diabetic skeletal muscle.

## Figures and Tables

**Table 1:** Effect of salbutamol on blood glucose level (mg/dL), body weight (g), and GN muscle (mg) weight in HFD/STZ-induced diabetic rats. Data were represented as mean ± SD (*n* = 6). Statistical significance was determined using one-way ANOVA with Tukey’s multiple comparisons post hoc test. Table depict *** p < 0.001 vs. control and *### p <* 0.001 vs. HFD/STZ and ns: non significant.

Parameters	Time Points	Control	Salbutamol	HFD/STZ	HFD/STZ + Salbutamol
Blood Glucose level (mg/dL)	0 week	124.83 ± 1.47	124.33 ± 1.21	124.66 ± 3.26	124.66 ± 2.94
	4 weeks	124.83 ± 1.94	125.16 ± 1.72	371.16 ± 14.90 ***	370.83 ± 15.43 ^ns^
Body weight (g)	0 week	185.16 ± 2.31	194.50 ± 3.08	184.50 ± 2.07	175.00 ± 3.03
	4 weeks	206.66 ± 2.80	226.66 ± 1.96	306.33 ± 3.01 ***	203.50 ± 1.51 ^###^
GN muscle weight (mg)	4 weeks	783.50 ± 2.88	804.66 ± 2.73	338.66 ± 2.50 ***	776.16 ± 6.76 ^###^

## Data Availability

The data supporting the findings of this study are available upon request from the corresponding author.

## Supplementary Materials

The following supporting information can be downloaded at https://www.mdpi.com/article/10.3390/pharmaceutics15082101/s1. Figure S1: Stack plot of representative 800 MHz one-dimensional ^1^H CPMG NMR spectra of rat GN muscle of rat samples of four study groups; Figure S2: Multivariate statistical analysis.
