# Supplementary material for: Salbutamol Attenuates Diabetic Skeletal Muscle Atrophy by Reducing Oxidative Stress, Myostatin/GDF-8, and Pro-Inflammatory Cytokines in Rats
Source: Pharmaceutics. 2023 Aug 8;15(8):2101. doi: 10.3390/pharmaceutics15082101 (PMC10458056; doi:10.3390/pharmaceutics15082101)
Supplement: Supplementary file 1 [file pharmaceutics-15-02101-s001.zip › pharmaceutics-2502134-supplementary.pdf]

Supplementary Material:

Salbutamol Attenuates Diabetic Skeletal Muscle Atrophy by Reducing Oxidative Stress, Myostatin/GDF-8, and Pro-inflammatory Cytokines in Rats

Anand Kumar [1], Priyanka Prajapati [1], Gurvinder Singh [2], Dinesh Kumar [2], Vikas Mishra [1], Seong-Cheol Kim [3], Chaitany Jayprakash Raorane [3,*], Vinit Raj [3,*] and Sapana Kushwaha [4,*]

[1] Department of Pharmaceutical Sciences, School of Pharmaceutical Sciences, Babasaheb Bhimrao Ambedkar University, Vidya Vihar, Raebareli Road, Lucknow 226025, India; anandkumarpharm@gmail.com (A.K.); priyankaprajapati243@gmail.com (P.P.); vikasmishra12@gmail.com (V.M.)

[2] Centre of Biomedical Research, SGPGIMS Campus, Lucknow 226014, India; gourav1752@gmail.com (G.S.); dineshcbmr@gmail.com (D.K.)

[3] School of Chemical Engineering, Yeungnam University, Gyeongsan 38541, Republic of Korea; sckim07@ynu.ac.kr

[4] National Institute of Pharmaceutical Education and Research (NIPER), Raebareli, New Transit Campus, Bijnor-Sisendi Road, Lucknow 226002, India

* Correspondence: chaitanyaraorane22@ynu.ac.kr (C.J.R.); drvinitraj@ynu.ac.kr (V.R.); sapana.kushwaha@niperrbl.ac.in (S.K.)

Supplementary Material:

Multivaiate analysis for fature screening:

Effect of salbutamol on skeletal muscle metabolomics using ^{1}H NMR-based technique in diabetic rats

Diabetes has its significant impact on metabolic profiles and treatment may restore these changes back to normal. Within this framework, we also performed the tissue/serum metabolomics analysis using 800 MHz NMR spectroscopy. The representative 1D ^{1}H NMR spectra of rat GN muscle samples acquired from the control group, salbutamol group, HFD/STZ group, and HFD/STZ + salbutamol group are stacked and have been shown in the electronic supplementary material **(ESM, Figure S1)**. The NMR peaks of different metabolites were identified as described previously (1) and have been annotated in the **Figure S1**. The NMR signals of various metabolites were identified such as (a) amino acids, *viz.* alanine, glycine, glutamate, glutamine, π-methylhistidine, leucine, isoleucine, phenylalanine, methionine, sarcosine, proline, threonine, serine, valine and tyrosine (b) energy metabolites, *viz.* acetate, creatine, fumarate, formate, glycerol, lactate, pyruvate, and succinate (c) lipoproteins (*viz.* VLDL and LDL) and (d) ketone bodies contents, *viz.* acetone, betaine, 3-hydroxybutyrate. Except lipid and membrane metabolits, the concentrations of other circulatory metabolites were estimated using NMR suite of CHENOMX software as described previously (2). Additionally, some relevant metabolic ratios were also estimated, *viz.* phenylalanine to tyrosine ratio (PTR), histidine to tyrosine ratio (HTR) and glutamate to glutamine ratio (EQR). In order to investigate if salbutamol treatment ameliorate the disturbed metabolic profiles in diabetic rats, the GN muscle metabolic profiles were compared using multivariate statistical analysis. This was done with the help of free web-based software Metaboanalyst (v4.0) (Xia & Wishart, 2016). The 3D score plot obtained from the PLS-DA analysis showed a distinct separation among the four groups, demonstrating a substantial metabolic disparity in the HFD/STZ and HFD/STZ + salbutamol groups with respect to the control group and the salbutamol group by itself (See ESM, Figure S2A). The relative closeness of samples of salbutamol treated groups compared to HFD/STZ groups (See ESM, Figure S1A) suggested the therapeutic response of salbutamol.

Further, the PLS-DA analysis was used to identify the metabolic features of discriminatory relevance based on the variable importance in projection (VIP) score values > 1.0 (See ESM, Figure S2B) and subseqeuntly confirmed through performing machine learning random forest

Supplementary Material:

(RF) classification analysis (See ESM, Figure S2C). The mean decrease accuracy (MDA) score plot derived from the RF clustering approach was used to confirm the discriminatory potential of metabolic profiles in classifying the data (See ESM, Figure S2D). The integrative analysis (based on VIP and MDA score plots and ANOVA statistics) identified several metabolic entities of discriminatory potential which were then evaluated for their statistical significance using the ANOVA test. The quantitative variations in these discriminatory features have been shown through box-cum-whisker plots (Figure 11) generated using ANOVA module of Metaboanalyst. Compared to control rats, the GN muscle levels of 3-hydroxybutyrate, sarcosine, succinate, HTR, PTR, and EQR were elevated, and creatine and glycine levels were decreased in the HFD/STZ group . As evident from the result (Fig. 10), several GN muscle metabolites showed a metabolic reprogramming trend suggesting salbutamol could potentially alleviate the metabolic alterations in diabetic rat muscle.

Supplementary Material:

Figure S1: Stack plot of representative 800 MHz one-dimensional 1H CPMG NMR spectra of rat GN muscle of rats samples of four study groups: control (blue), salbutamol (red), HFD/STZ (green) and HFD/STZ + Salbutamol (purple). The spectral peaks of specific metabolites are labelled as per their resonance assignment confirmed using CHENOMX profiler. Key acronyms are: HDL: high-density lipoprotein; LDL: low density lipoproteins; VLDL: very-low density lipoproteins; NAG: N-acetyl glycoproteins; 3HB: 3-hydroxybutyrate; GPC: glycerophosphocholine; TMAO: trimethyl-amine N-oxide; N,N-DMgly: N,N-Dimethylglycine; Phe: phenylalanine; Leu/Lys: leucine/lysine and UI: unidentified.

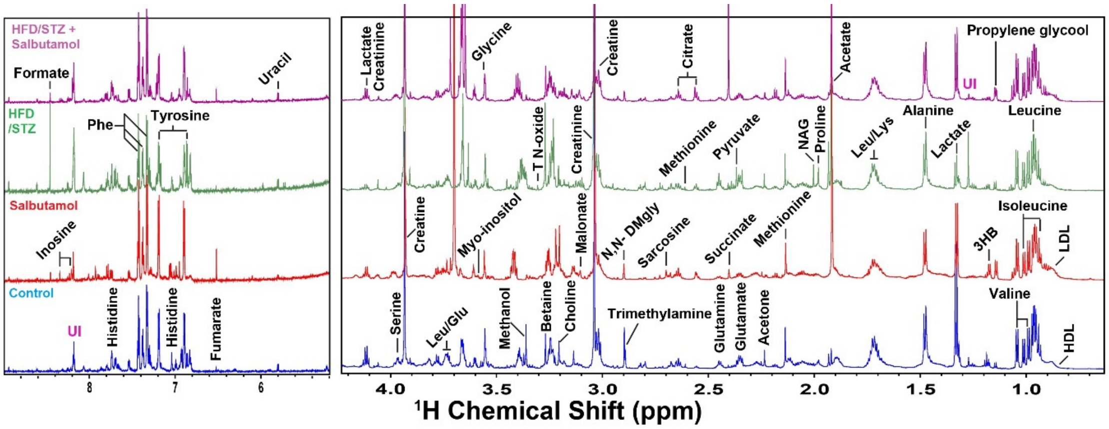

Supplementary Material:

Figure S2: Multivariate statistical analysis. **(A)** PLS-DA based 3D score plot derived from the concentration profiles of GN muscle metabolites estimated with respect to format. **(B)** The cross validation of PLS-DA model and bar-graph showing the performance based on 1 to 5 PLS-DA model components. **(C)** The VIP score plot based on PLS-DA model showing indexing of metabolites based on their discriminatory potential and **(D)** the mean decrease accuracy (MDA) score plot derived from machine learning random forest clustering approach showing important metabolic features exhibiting both discriminatory potential and statistically significant difference in different groups. Key acronyms are: 3-HB: 3-hydroxybutyrate; HTR: histidine to tyrosine ratio; PTR: phenylalanine to tyrosine ratio; EQR: glutamate to glutamine ratio.

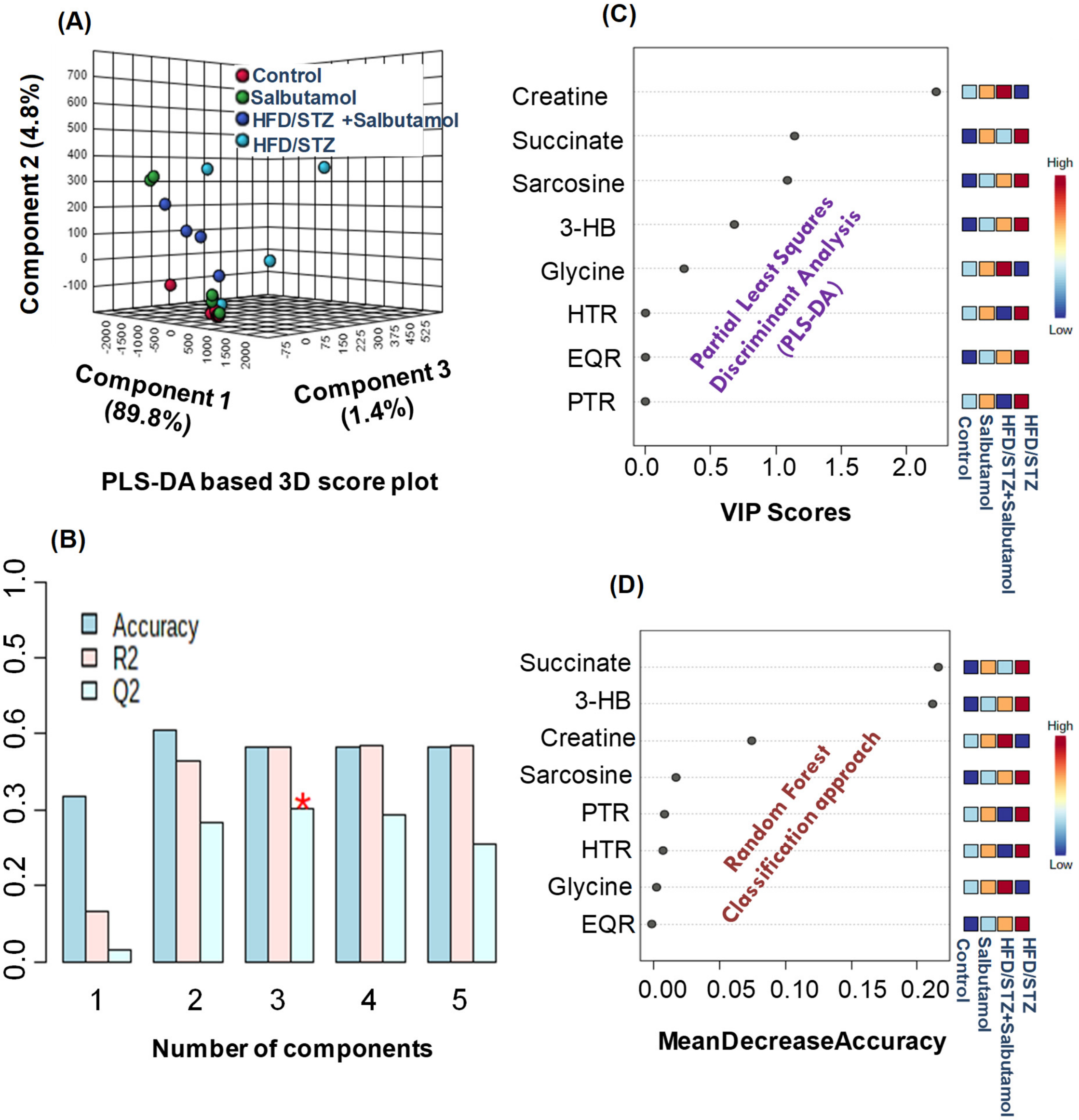

Supplementary Material:

References:

(1) Guleria, A., Pratap, A., Dubey, D., Rawat, A., Chaurasia, S., Sukesh, E., Khetrapal, C. L. (2016). NMR-based serum metabolomics reveals a distinctive signature in patients with Lupus Nephritis. *Scientific reports, 6*(1), 1-11.

(2) Singh, A., Prakash, V., Gupta, N., Kumar, A., Kant, R., & Kumar, D. (2022). Serum Metabolic Disturbances in Lung Cancer Investigated through an Elaborative NMR-Based Serum Metabolomics Approach. *ACS Omega*, 7, 6, 5510–5520.
